# Phase Separation and Elastic Properties of Poly(Trimethylene Terephthalate)-*block*-poly(Ethylene Oxide) Copolymers

**DOI:** 10.3390/polym8070237

**Published:** 2016-06-23

**Authors:** Elżbieta Piesowicz, Sandra Paszkiewicz, Anna Szymczyk

**Affiliations:** 1 Institute of Material Science and Engineering, West Pomeranian University of Technology, Piastow Av. 19, 70-310 Szczecin, Poland; senel@zut.edu.pl; 2 Institute of Physics, West Pomeranian University of Technology, Piastow Av. 19, 70-310 Szczecin, Poland; anna.szymczyk@zut.edu.pl

**Keywords:** poly(ether-ester), poly(trimethylene terephthalate), phase separation, elastic properties, thermoplastic elastomer

## Abstract

A series of poly(trimethylene terephthalate)-*block*-poly(ethylene oxide) (PTT-*b*-PEOT) copolymers with different compositions of rigid PTT and flexible PEOT segments were synthesized via condensation in the melt. The influence of the block length and the block ratio on the micro-separated phase structure and elastic properties of the synthesized multiblock copolymers was studied. The PEOT segments in these copolymers were kept constant at 1130, 2130 or 3130 g/mol, whereas the PTT content varied from 30 up to 50 wt %. The phase separation was assessed using differential scanning calorimetry (DSC) and dynamic mechanical thermal analysis (DMTA). The crystal structure of the synthesised block copolymers and their microstructure on the manometer scale was evaluated by using WAXS and SAXS analysis. Depending on the PTT/PEOT ratio, but also on the rigid and flexible segment length in PTT-*b-*PEO copolymers, four different domains were observed i.e.,: a crystalline PTT phase, a crystalline PEO phase (which exists for the whole series based on three types of PEOT segments), an amorphous PTT phase (only at 50 wt % content of PTT rigid segments) and an amorphous PEO phase. Moreover, the elastic deformability and reversibility of PTT-*b*-PEOT block copolymers were studied during a cyclic tensile test. Determined values of permanent set resultant from maximum attained stain (100% and 200%) for copolymers were used to evaluate their elastic properties.

## 1. Introduction

Polyester thermoplastic elastomers (TPE) have been known since the 1970s by the trade name Hytrel. Until recently, *t*he majority of these commercially available poly(ether-ester) block copolymers with elastic properties consisting of crystallisable butylene terephthalate sequences (rigid polyester segments) and poly(alkylene oxide) sequences (flexible polyether segments) were produced based on the petrochemical monomers [1]. Depending on the polyether/polyester block ratio, poly(ether-ester) copolymers are manufactured with a wide range of mechanical and biodegradation behaviour. The growing interest in eco-friendly products creates an increased demand for production of TPEs which will be partially or fully based on monomers from renewable sources. In recent years, after the development of production of bio-based 1,3-propanediol (PDO), lactic acid from starch-derived glucose, and 1,4-butanediol (BDO) using Genomatica’s technology, growing production of polyesters and thermoplastic elastomers [2,3,4] based on these renewable source monomers has been observed. Currently, several types of bio-based TPEs based on poly(butylene terephthalate) (PBT) as rigid segments are commercially produced, such as Arnitel^®^Eco (DSM Engineering Plastics, Geleen, Netherlands) and Hytrel^®^RS (DuPont). Moreover, Hytrel^®^RS, thermoplastic polyester elastomer, contains flexible segments made form polyether (Cerenol™, Pascagoula, MS, USA) derived from bio-PDO [5]. The partially or fully bio-based aromatic polyesters such as poly(trimethylene terephthalate) (PTT) [6,7], poly(butylene furanoate) (PBF) [8], poly(trimethylene furanoate) (PTF) [9] and biodegradable bio-based biodegradable aliphatic polyesters such as poly(lactic acid) (PLA) [10], poly(caprolactone) (PCL) [11] and poly(butylene succinate) (PBS) [12,13] have recently been investigated as promising building blocks in TPEs, especially for medical applications [14,15]. Among them, the poly(ethylene oxide) (PEO)-based block copolymers are also of interest for medical applications due to their superior biocompatibility and biostability [10]. Because of their high mobility, amorphous PEO chains are used to act as the flexible blocks appearing along the copolymeric backbone [16]. Cohn et al. [16,17,18,19] synthesized poly(ethylene oxide)/poly(lactic acid) (PEO/PLA) [17,18,19] and poly(ethylene oxide)/poly(caprolactone) (PEO/PCL) [20] multiblock copolymers and investigated their properties. It was confirmed that PEO/PLA (PELA) copolymers performed most satisfactorily as films for the prevention of post-surgical adhesions in various animal models [21,22]. Moreover, PEO-based copolymers were identified as promising for the design of CO_2_ selective membranes [23,24]. They were found to have high CO_2_ permeability and selectivity, since the ether linkage with two electron pairs, favorably interacts with CO_2_ due to dipole-quadrupole interactions [25,26]. Copolymers or block copolymers containing polyether segments are interesting, especially those based on polyethylene oxide (PEO), which were already studied for gas separation membranes [27,28,29]. In our previous work [30], the design and synthesis of poly(trimethylene terephthalate)-*block*-poly(ethylene oxide) copolymers (PTT-*b*-PEOT) with well-defined properties (tailor-made) were reported. Moreover, the relationship between gas transport properties and physical properties of PTT-*b*-PEOT has been also discussed. 

In developing elastomers for medical applications, it is important to design elastomers having both satisfactory mechanical properties and biodegradability. Elastomers based on biodegradable aliphatic polyesters exhibit poor physical and mechanical properties in some applications. Partially bio-based PTT have excellent physical and mechanical properties compared to aliphatic polyesters, but like other terephthalate polyesters, possess strong resistance to bacterial or fungal attack, which results in their low degradability under the environmental conditions. Therefore, thermoplastic elastomers based on PTT as a rigid segment and PEOT as flexible segment having both satisfactory mechanical properties and biodegradability can be useful in some medical applications. Previously reported research on PTT based block copolymers has shown that the elastic properties and gas-transport properties of this group of materials can be tailored during polymer synthesis by altering the molecular weight of flexible polyether blocks and the amount of rigid polyester block or by introduction of nanofillers [6,30,31]. Moreover, the group of Yao et al. [32,33,34] studied the influence of PTT-*b*-PEOT copolymers’ composition on their crystallization behavior. However, no study was presented on the influence of the composition on the mechanical, especially elastic properties resulting from the heterophase structure. The present work focuses on the characterization of mechanical properties of PTT-*b*-PEOT thermoplastic elastomers. The aim of the study was to investigate the influence of PEO content and segment length in the block copolymer on the elastic properties. Following a two-step synthesis, PTT-*b*-PEOT were synthesized, whereby a family of bio based poly(ether-ester) multiblock copolymers was obtained. The PEG with molecular weight of 1000, 2000, 3000 g/mol was used to build blocks for flexible/soft segment (PEOT). Herein, light will be shed on the relationship between their composition, phase separation and elastic properties.

## 2. Experimental Section

### 2.1. Synthesis of PTT-Block-PEOT Copolymers

**Figure 1:**
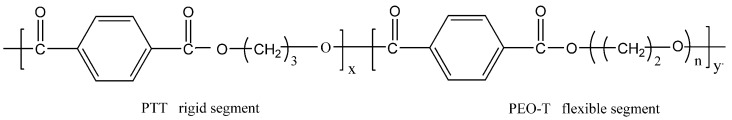
Chemical structure of poly(trimethylene terephthalate)-*block*-poly(ethylene oxide) copolymers.

The PTT-*b*-PEOT copolymers were synthesized by a two-step method comprised of transesterification and polycondensation reaction of dimethyl terephthalate (DMT, Sigma-Aldrich, Poznań, Poland), bio-1,3-propanediol (PDO, Susterra^®^Propanediol, DuPont Tate & Lyle, City, US State, USA) and poly(ethylene glycol) (PEG, Sigma-Aldrich) with molecular weight of 1000 (P1), 2000 (P2) and 3000 (P3) g/mol in the presence of titanium (IV) butoxide (Fluka) as catalyst according to the method described elsewhere [7,30]. Irganox 1098 (Ciba-Geigy, Basel, Switzerland) was used as thermal stabilizer. The chemical structure of synthesized copolymers is presented in Figure 1 and their composition and molecular weight are collected in Table 1. The synthesized copolymers are random copolyesters (PTT units and PEO-T units). The average length “x” of the rigid PTT segment was calculated based on the conventional flexible segments definition, which includes one terephthalate unit (T) with each PEO sequence, called PEO-T, as illustrated in Figure 1.

### 2.2. Sample Preparation

The dumbbell shape samples (type A3) for tensile tests and dynamic-mechanical thermal analysis (DMTA) measurements were obtained by injection moulding using Boy 15 (Dr BOY GmbH & Co., Neustadt-Fernthal, Germany) injection moulding machine with the following parameters: injection pressure 55 MPa, temperature 15 °C higher than the melting point determined by DSC, mold temperature 30 °C, holding down pressure of 20 MPa for 15 s and cool time of 10 s.

### 2.3. Characterization Methods

The thermal properties of the synthesized copolymers were measured with differential scanning calorimetry (DSC, TA Instrument Q-100, New Castle, DE, USA) in a heating-cooling-heating cycle from −100 to 250 °C. The first cooling and second heating scans were used to determine the melting and crystallization peaks. The heating and cooling rate was 10 °C/min and sample size approximately of 10 mg. The glass transition temperature (*T*_g_) for the polymer samples was taken as the midpoint of the change in heat capacity. The degree of crystallinity of PEO and PTT were calculated by the following equation: *x_c_* = (Δ*H*_m_/$\Delta H_{m}^{o\;}$); where $\Delta H_{m}^{o}$ is the enthalpy change of melting for a 100% crystalline material and Δ*H*_m_ is derived from the melting peak area on a DSC thermogram. The enthalpy values of 197 J/g [30] and 146 J/g [35] were used for 100% crystalline PEO and PTT, respectively.

The DMTA has been performed using a Polymer Laboratories MK II apparatus working in a bending mode in a temperature range from −100 °C to the polymer melting temperature, at a frequency of 1 Hz and the heating rate of 3 °C/min. The storage modulus (*E*′), loss modulus (*E*′′) and tan δ were determined as a function of temperature with the maximum (β) of the E′′ and tan δ taken as glass transition.

Small angle X-ray scattering (SAXS) measurements were performed at beam line A2 at HASYLAB (DESY, Hamburg, Germany) with a monochromatic radiation of wavelength 0.15 nm. Sample to detector distance calibration and beam centre was done using dry rat tail tendons. Long period (L) values was calculated from the Lorenz corrected SAXS intensity trough L = 1/s_max_, where s = 2πsinθ/λ is the scattering vector, and 2θ is the scattering angle.

Wide-angle X-ray scattering (WAXS) measurements of the synthesized copolymers were carried out using a X’Pert PRO diffractometer (PANalytical, Almelo, the Netherlands). The CuKα radiation (λ = 0.154 nm) was used generated using an applied voltage 40 kV. The spectra were registered in the 2-theta range of 4 to 40° with a step 0.05°.

Tensile measurements were performed at room temperature (23 °C) using an Instron 5566 universal tensile testing frame, equipped with a 5 kN Instron load cell, an contact optical long travel extensometer (Zwick, Ulm, Germany) and the Bluehill 2 software (Instron, Norwood, MA, USA), following the same procedure as that described for PTT-PTMO block copolymer [6,31]. Before the measurement, dumbbell shape samples were conditioned for eight weeks at 23 °C and the relative humidity of 50% ± 5%. Mean values and standard deviations were calculated for tensile modulus, stress and strain at break. Moreover, in order to investigate the elastic deformability and reversibility of the synthesized copolymers in tension, a previously reported [6] procedure has been applied. The procedure was based on the cycling loading and unloading of the specimen. The arbitrary chosen deformation speed was 100 mm/min. A confidence interval for the mean values of tensile strength and permanent set was then calculated according to the ISO 2854 standard. 

## 3. Results and Discussion

### 3.1. Phase Separation and Structure of PTT-b-PEOT Copolymers

In block copolyesters, due to the thermodynamic immiscibility of the rigid and flexible segments during cooling from the melt or solvent evaporating, localized micro/nano-phase separation occurs, leading to a well-organized domain structure with the final bulk material properties strongly dependent on the extend microphase separation and by the morphological characteristics of the domains [1,6]. The semicrystalline microphase (hard domains) created by segregation of rigid segments act as physical crosslinks, playing a role similar to chemical crosslinks in vulcanizates and imparting the material’s elastomeric behaviour. 

**Figure 2:**
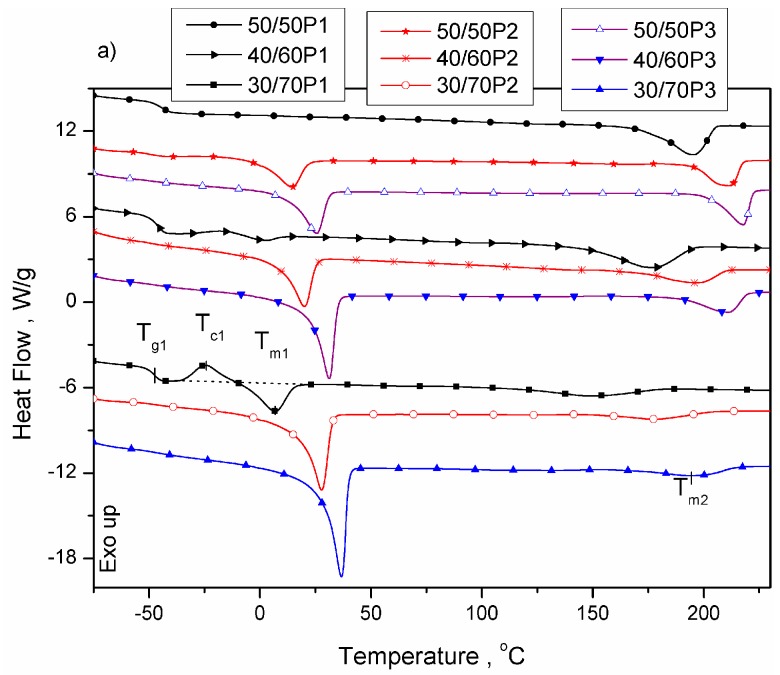

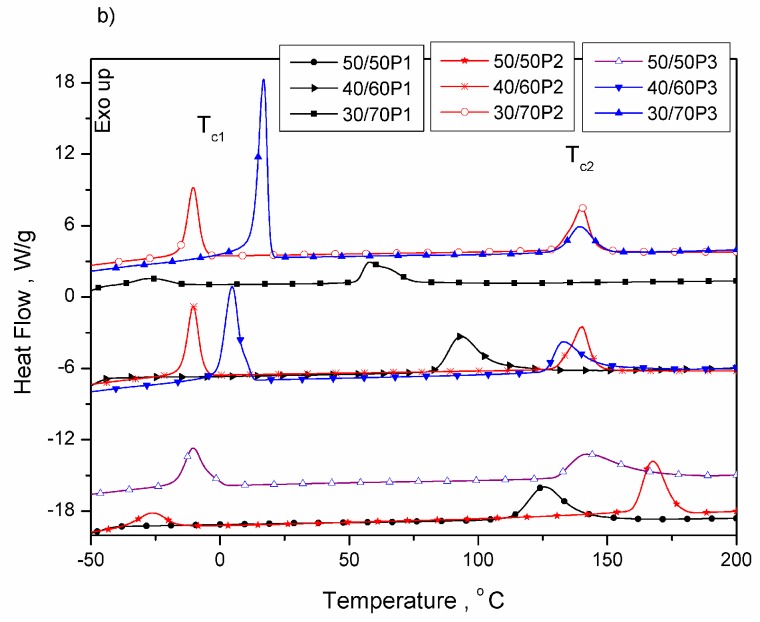
DSC thermograms obtained during 2nd heating (**a**) and cooling (**b**) for PTT-*b*-PEOT copolymers.

In our previous paper, it was confirmed [7] that, in PTT-*b*-PEOT, copolymer separates into a semicrystalline PTT hard phase and a PEO-rich amorphous soft phase. Herein, the effect of the flexible polyether (PEOT) segment length (1130 (P1), 2130 (P2), 3130 (P3) g/mol) on the microseparated phase structure of the synthesized PTT-*b*-PEOT block copolymers was investigated by using the DSC and DMTA. Based on WAXS and SAXS analysis, the crystal structure of the block copolymers and their microstructure on the manometer scale was evaluated. The DSC thermograms of the copolymers are shown in Figure 2, and the results are summarized in Table 2. It is already known that PEO sequences with molecular weight above 600 g/mol are able to crystallize and for PEOT segments above 2000 g/mol, the melting point is higher than room temperature [1]. Hence, at room temperature, the presence of PEO crystals beside PTT crystals dispersed in amorphous soft PEO-rich phase in microseparated phase structure of the synthesized PTT-*b*-PEOT block copolymers can influence the elastic properties of the obtained copolymers. As the PEOT segment length increases in PTT-*b*-PEOT copolymers, the phase miscibility decreases. It was confirmed by two remarks: (1) the slight shift of glass transition temperature (*T*_g1_) of PEO-rich soft phase in the lower temperature region for sample 30/70P1 and (2) independence of *T*_g1_ (~−48 °C) on the PTT/PEOT ratio in block copolymers with longer PEOT flexible segments (P2, P3). For copolymers containing higher content of flexible PEOT segments with *M*_w_ of 1130 g/mol (sample 30/70P1) or longer PEOT flexible segments lengths (2130 or 3130 g/mol), both melting and crystallization temperatures (*T*_m1_, *T*_c1_) of PEO sequences have been noted (Table 2). Crystalline PEO domains were observed in PTT*-b*-PEOT multiblock copolymers containing PEOT segments with *M*_w_ of 2130 and 3130 g/mol and for copolymers containing 70 wt % of PEOT segments with *M*_w_ of 1170 g/mol. The content of PEO crystallites ($x_{c}^{\mathrm{PEO}\;}$) and melting temperature (*T*_m1_) increase with increasing segment length and content of PEOT segments in copolymers. During cooling from the melt (after crystallization of PTT sequences), the PEO crystals were formed at higher temperatures (*T*_c1_) in copolymers containing PEOT segments with *M*_w_ of 3130 g/mol than for copolymers with PEOT segments with *M*_w_ of 2130 g/mol. It is known that the increasing chain length leads to an increase in the equilibrium melting temperature, *T*_m_, hence, to an increase in the supercooling, ∆*T* = *T*_m_ – *T*_c_, and in the driving force for crystallization. These competing effects result in the classical bell-shaped curve of spherulitic growth rate or crystallization rate vs. chain length, which were observed for polymers such as PEO [36], or poly(є-caprolactone) [37]. Analysis of the degree of supercooling (∆*T*_PEO_, ∆*T*_PTT_, in Table 2) for PEO and PTT crystal growth showed that the length of 2130 g/mol for PEOT segment can be optimal in PTT-*b*-PEOT block copolymer in comparison with copolymer containing PEOT with molecular weight of 3130 g/mol. In these systems, the lowering of the degree of supercooling for PEO crystallization, with the increasing content of PEOT segments in block copolymer, indicate that an increase of PEO crystallization rate is observed. The lower values of the degree of supercooling for PTT crystallization also indicate the increase of PTT crystal growth rate in these systems. It was confirmed by higher crystallization and melting temperatures (*T*_c2_, *T*_m2_) of PTT crystals and an increase in the fusion (crystallization) heat of PTT segments (Table 2, Figure 2). The content of PTT crystals decreases with the increasing content of PEOT in the block copolymer. However, copolymers with longer PEOT flexible segments exhibited higher or comparable (for samples 50/50P2 and 50/50P3) content of PTT crystals at a constant PTT/PEOT ratio.

**Figure 3:**
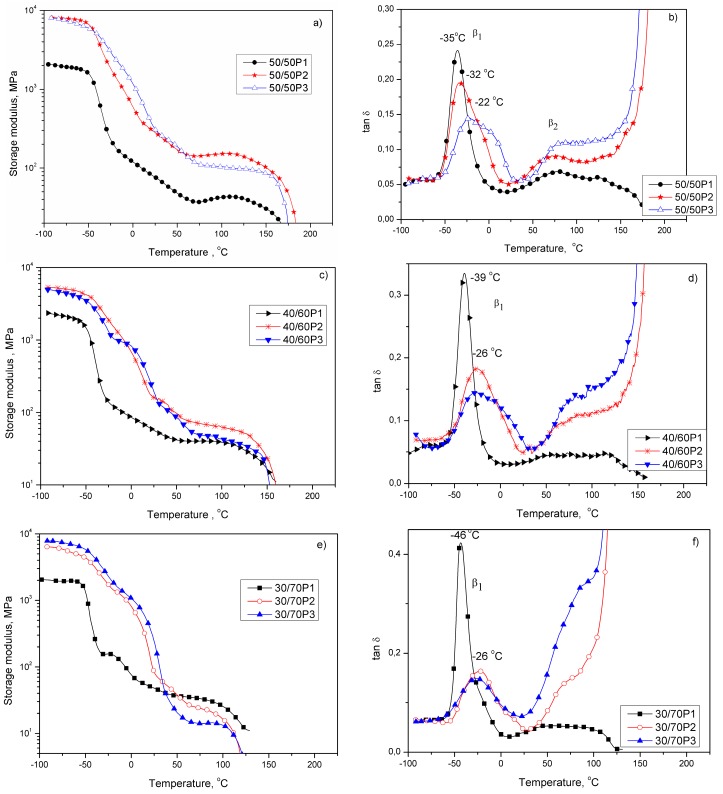
The storage modulus (**a**,**c**,**e**) and tan δ (**b**,**d**,**f**) as a function of temperature for PTT-*b*-PEOT copolymers with PEOT content of 50 (**a**,**b**), 60 (**c**,**d**) and 70 (**e**,**f**) wt %.

The influence of PEOT flexible segment length on phase separation in block copolymers was also evaluated by DMTA. Changes of the storage modulus (*E*’) and loss tangent (tan δ) as a function of temperature are presented in Figure 3. The DMTA analysis confirmed that PTT-*b*-PEOT copolymers are multiphase systems, and the number of phases is dependent on the block copolymer composition and segment length. The first drop of the storage modulus (*E*’) at low temperature range is related to the glass transition of the amorphous soft polyether phase (Figure 3a,c,e). The second drop of the *E*’ is due to the softening point of the hard phase (melting of PTT crystals), which is dependent on the composition and appears around 120–180 °C, 110–150 °C and 90–125 °C for copolymers with the PTT/PEO ratio of 50/50, 40/60 and 30/70, respectively (Figure 3a,c,e). Block copolymers containing PEOT with higher molecular weight (2130 and 3130 g/mol) at a constant PTT/PEOT ratio (Figure 3a,c,e) show higher values of storage modulus than copolymers containing PEOT segments with molecular weight of 1130 g/mol due to the presence of PEO crystallites beside PTT crystallites (confirmed by DSC results). The copolymers with the PTT/PEO ratio of 30/70 displayed a clearly visible shoulder in the storage modulus after glass transition at temperature range from −25 to 40 °C, due to the presence of the PEO crystalline phase that melts in this temperature range. The presence of two β-relaxations, β_1_ and β_2_, on the tan δ curves (Figure 3b) for block copolymers containing the highest content (50 wt %) of PTT rigid segments is observed. The β_1_-relaxation present in low-temperature region corresponds to the amorphous PEO-rich phase (or amorphous PEO/PTT blended phase), and the β_2_-relaxation present at around 70 °C is due to the amorphous phase of semicrystalline PTT domains (Figure 3b). As the PTT content increases, the rigid segment length also increases, as shown in Table 1, and the longer rigid segments display a greater tendency to form a more highly organized separated phase (semicrystalline PTT domains) in preference to mixing with the soft phase. The β_2_-relaxation is not present on tan δ curves for copolymers containing a lower content of PTT rigid segments in block copolymers. It is known that the glass transition temperature of the PTT amorphous phase (40 °C) [6,38] appears at a temperature close to the melting temperature of PEO crystals, hence the melting process of PEO crystals can interfere with the observation of the glass transition temperature of an amorphous phase in semicrystalline PTT domains at lower content of PTT segments in the copolymers. These perhaps are the main reasons why the glass transition temperature of amorphous PTT segments was not detected in DSC measurements.

The maximum of β_1_-relaxation, which corresponds to *T*_g1_ on DSC curves, for all PTT-*b*-PEOT copolymers, broadened and shifted to higher temperatures (from −46 to −26 °C) with an increase of flexible segment length. This behavior, but also the broadening of β_1_-relaxation region, can be explained as a combination of a variety of processes probably related to the segment length distribution of flexible and rigid ones. The shift of the maximum of β_1_-relaxation toward higher temperatures may result from mixing of uncrystallized PTT with PEO units in amorphous phase, which causes a decrease in its thermal molecular mobility. Moreover, the decrease of β_1_-relaxation intensity and its shift toward higher temperatures at constant PTT/PEOT ratio for copolymers with increasing length of PEOT flexible segments can result not only from the increasing content of uncrystalized PTT sequences in amorphous PEO-rich phase but also from the increasing content of semicrystalline PEO domains distributed in amorphous PEO phase. A similar effect of the flexible segment (PEO) length on the microphase separation in segmented copolymers based on poly(ethylene oxide) with high molecular weight of 2000 and 6000 g/mol, and on poly(ethylene terephthalate) (PET), was observed by Wang et al. [39]. They observed that PEO segments in a segmented block copolymer showed a higher glass transition temperature (*T*_g1_), lower melting temperature (*T*_m1_) and crystallinity than the corresponding PEO homopolymer due to the reduction of the thermal molecular motion of PEO segment effectively restricted by the phase mixing between rigid (PET) and flexible PEOT segments. Both long PEO segment and high PET content lead to a high degree of microphase separation in the segmented PET-*b*-PEOT copolymers. The rigid segment’s ability to quickly produce the crystal structure during processing in these types of elastomers is a necessary requirement. Using PET as the rigid segment is accompanied by certain limitations related to its slower crystallization rate than that of PTT or PBT [38].

**Figure 4:**
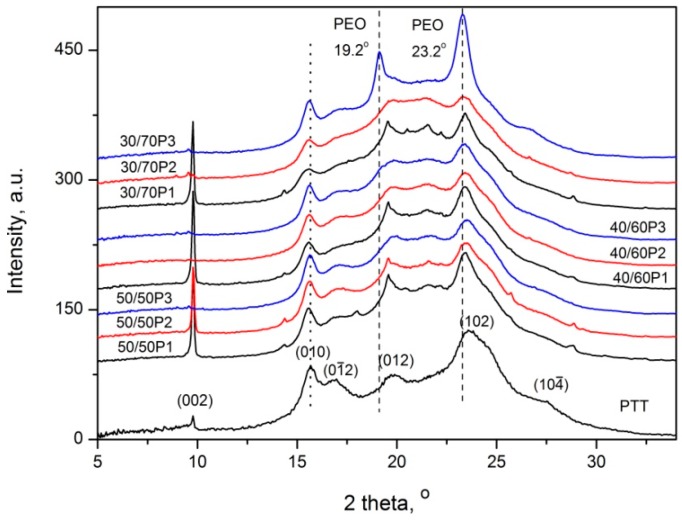
WAXS patterns of PTT homopolymer and PTT-*b*-PEOT copolymers at 23 °C.

WAXS and SAXS measurements confirmed the results obtained by DSC and DMTA that, in PTT-*b*-PEOT copolymers containing PEOT segments with molecular weight of 2130 and 3130 g/mol at room temperature, PTT and PEO crystals can be present. Figure 4 presents WAXS patterns of neat PTT homopolymer and PTT-*b*-PEOT copolymers. All of the diffractograms of the block copolymers exhibited major characteristic crystalline peaks at the scattering angles 2θ of *ca.* 9.7°, 15.6°, 16.7°, 19.6°, 23.6° and 27.4°, which are related to PTT crystals and correspond to the reflection planes of (002), (010), $\left(0\bar{1}2\right)$, (012), (102), (10$\bar{4}$) [6]. PEO crystals possess two characteristic peaks at 19.2° and 23.3°, which correspond to interplanar distances of 0.46 and 0.38 nm, respectively [40]. These peaks in PTT-*b*-PEOT block copolymers can overlap with peaks of PTT crystals. For block copolymers containing PEOT segments with molecular weight of 1130 g/mol, the crystal lattices of the block copolymer do not change. This can indicate that only one PTT crystal structure exists in these copolymers. The shift of the intensity of peak at 19.6° and 23.6° to the position of 19.2° and 23.3° for block copolymer containing the highest content (70 wt %) of PEOT segments with molecular weight of 3130 g/mol confirms that, in this copolymer, the PEO crystals coexist with PTT crystals.

**Figure 5:**
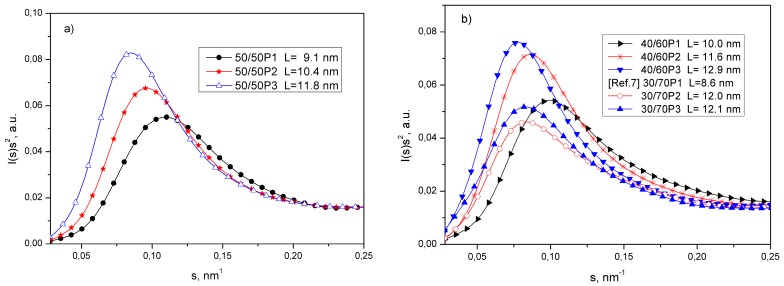
The Lorentz–corrected SAXS patterns at 23 °C (L—long period) for PTT/PEOT ratio of (**a**) 50/50 and (**b**) 40/60 and 30/70.

Figure 5 presents the Lorentz corrected SAXS patterns for PTT-*b*-PEOT copolymers. It can be seen that SAXS curves exhibit a distinct diffraction maxima confirming that PTT or PEO crystallizes into a distribution of lamellar crystals separated by amorphous regions. It can be seen that the calculated values of the long periods (L presented in Figure 5), which is the sum of the average thicknesses of the crystalline lamella and the amorphous region, increase with the increased length and content of PEOT segment in the block copolymer.

The increase of long periods for copolymers containing 50 wt % of PEOT flexible segments can be due to the increase of the crystalline layer thickness of PTT crystals. DSC results confirmed (Table 2) that, in these copolymers at room temperature, only crystals of PTT are present. For block copolymers containing PEOT segments with molecular weight of 3130 g/mol, the observed diffraction maxima can be attributed to the mixture of PEO and PTT lamellar crystals dispersed in an amorphous soft phase and the determined values of long period represent an average value of both of the lamellar structures.

Summing up the above mentioned results, one can conclude that the structure of the herein investigated PTT-*b*-PEOT copolymers resembles the supramolecular structure of block copolymers based on PET, PBT and PTT as rigid segments and PTMO and PEO as flexible ones [6,7,41,42,43]. Depending on the PTT/PEO ratio but also on the rigid and flexible segment length in PTT-*b-*PEO copolymers, four different domains were observed i.e., a crystalline PTT phase, a crystalline PEO phase (which exists for the whole series based on P2, P3, and 30/70P), an amorphous PTT phase (only at 50 wt % content of PTT rigid segments) and an amorphous PEO phase.

### 3.2. Elastic Properties of PTT-b-PEOT Copolymers

**Figure 6:**
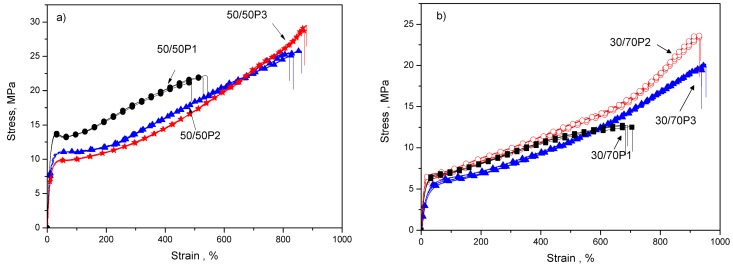
Representative stress–strain curves for PTT-*b*-PEOT copolymers containing 50 (**a**) and 70 (**b**) wt % of PEOT flexible segments.

The representative stress–strain curves of two series of the PTT-*b*-PEOT copolymers with the lowest (50 wt %) and highest (70 wt %) content of PEOT flexible segments are presented in Figure 6. The presented curves obtained during uniaxial tensile tests have a typical course of thermoplastic elastomer [41]. Moreover, the values of tensile modulus (*E*), stress at break (σ_b_) and strain at break (ε_b_) for the whole series of block copolymers are summarized in Table 3. Generally for poly(ether-block-ester) copolymers containing non-crystallisable flexible polyether segments [7,35], as a consequence decreasing the number of polyester crystalline domains that contribute to the strength, the decrease in tensile modulus and tensile strength at a break with an improvement in elongation at a break with increasing the polyether (PTMOT, PEOT) flexible segment content in the block copolymer was observed [1,7,35]. Such behavior is confirmed for the synthesized series of block copolymers based on PEG with molecular weight of 1000 and 2000 g/mol when tensile modulus and stress at break decreased with increasing of flexible PEOT segment content in copolymer. In turn, for PTT-b-PEOT copolymers based on PEG with molecular weight of 3000 g/mol, an increase in the tensile modulus with an increase of PEOT content in the block copolymer has been noticed. It is well known that the tensile modulus increases with an increase of degree of crystallinity for semicrystalline polymers. The increase of modulus and higher values of tensile strength at break for copolymers with longer (2130 and 3130 g/mol) PEOT flexible segments, but at constant PTT/PEOT ratio, can be attributed to the presence of PEO crystallites (at room temperature), beside PTT crystalline domains (confirmed by DSC and DMTA), which can act as additional crosslinkers. On the other hand, values of elongation at break increase with an increase of PEOT flexible segments in copolymers but also with an increase of the flexible segment length. It can result from the presence of longer soft segment chains, and, as mentioned above, higher content of interphase.

**Figure 7:**
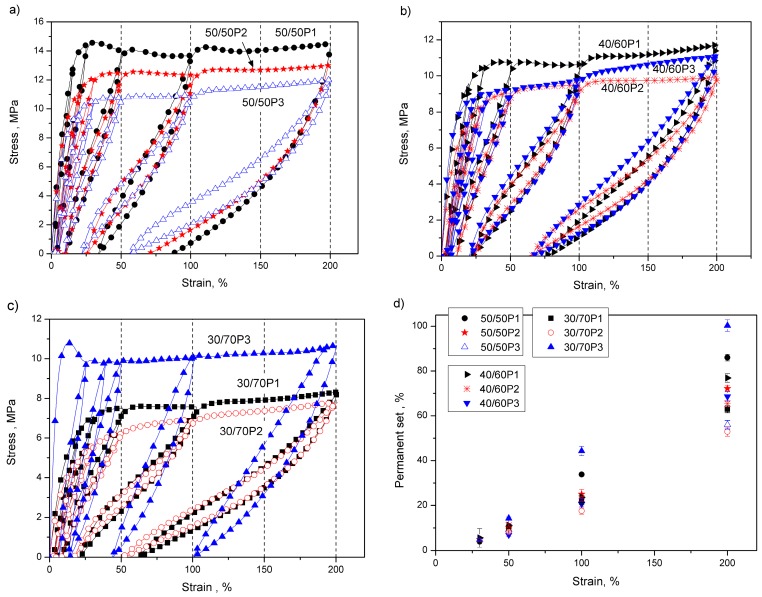
Stress–strain curves from cyclic tensile tests obtained for: (**a**) PTT-*b*-PEOT 50/5; (**b**) PTT-*b*-PEOT 40/60 and (**c**) PTT-b-PEOT 30/70 with PEG molecular weights of 1000, 2000 and 3000 g/mol and (**d**) permanent set of specimens in tension direction resultant from maximum attained strain in the cyclic deformation for PTT-*b*-PEOT copolymers.

Besides the uniaxial tensile tests, the loading–unloading properties of the synthesized PTT-*b*-PEOT copolymers were investigated to reveal the elastic properties. Measurements of stress–strain relationships in a cyclic loading and retraction, obtained for copolymers containing 50, 60, and 70 wt % of PEOT flexible segment with molecular weights of 1130 g/mol (series P1), 2130 g/mol (series P2) and 3130 g/mol (series P3), are shown in Figure 7. The investigated specimens exhibit a general characteristic common for thermoplastic block copolymers [6,40] that include a considerable permanent set and a change in the stress–strain relation every time a new maximum strain limit is achieved by the sample. Elastic recovery after established deformation was evaluated by determining the permanent set for the synthesized copolymers. It can be seen in Figure 7d that, for all copolymers, independently on PTT/PEOT ratio and length of PEOT flexible segment at low elongation (30 %), the values of residual strain are comparable to one another. However, the length of PEOT segment in the block copolymer at constant PTT/PEOT ratio has an important influence on their recovery from higher elongations (50%, 100%, 200%). The comparison of the values of the permanent set (Figure 7d) for copolymers with shorter and longer PEOT flexible segments in the block copolymer has shown that the best recovery properties (the lowest value of permanent set) exhibit copolymers containing PEOT flexible segments with molecular weight of 2130 g/mol at PTT/PEOT ratio of 30/70 and 40/60. One can see that copolymers containing PEOT with molecular weight of 3130 g/mol at PTT/PEOT ratio of 30/70 showed the highest values of permanent set, probably due to the highest content of hard phase (PEO and PTT crystals).

## 4. Conclusions

In conclusion, three series of PTT-*b*-PEOT copolymers were synthesized by a two-stage polymerization method that consists of transesterification and polycondensation in the melt. The length and content of flexible (PEOT) and rigid (PTT) segments in the polymer chain have been changed in order to observe the phase separation. All samples exhibited the microphase structure, which has been verified by the appearance of glass transition temperature (*T*_g1_) of soft phase in low temperature range and two melting points (*T*_m1_, *T*_m2_). Both PTT/PEO ratio and the rigid and flexible segment length affect the microphase structure. Thus, four different domains were observed i.e.,: a crystalline PTT phase, a crystalline PEO phase, an amorphous PTT phase (only at 50 wt % content of PTT rigid segments) and an amorphous PEO-rich phase. This phase separated structure is mainly responsible for the elastic properties of PTT-*b*-PEOT copolymers. The best recovery properties i.e., the lowest values of the permanent set, showed copolymers containing PEOT flexible segments based on PEG with molecular weight of 2000 g/mol and at PTT/PEOT ratio of 40/60 and 30/70. The synthesized block copolymers based on bio-propanediol (bio-PDO) and poly(ethylene glycol) (PEG) exhibit a good combination of mechanical properties as they are melt-processable and have good elasticity, which make them very interesting for application as thermoplastic elastomers, either in pure state and as a matrix for nanocomposites. 

## Figures and Tables

**Table 1:** Composition and molecular weight of synthesized PTT-*b*-PEOT copolymers.

Sample	*x*	*w*_PEOT_	$M_{w}^{\mathrm{PEOT}}$	*M*_n_ × 10^4^ by SEC	*M*_w_*/M*_n_
	mol	wt %	g/mol	g/mol	
50/50P1	5.48	50	1130	7.38	2.15
40/60P1	3.65	60	1130	7.79	2.23
30/70P1	2.35	70	1130	8.88	2.19
50/50P2	10.34	50	2130	7.69	1.69
40/60P2	6.88	60	2130	8.47	2.05
30/70P2	4.43	70	2130	9.64	1.90
50/50P3	15.20	50	3130	8.41	1.68
40/60P3	10.12	60	3130	10.05	1.63
30/70P3	6.50	70	3130	11.05	1.66

*x*—degree of polymerization of PTT segment with reference to 1 mol of PEOT segment; *w_PEOT_*—weight fraction of PEOT segments; $M_{w}^{\mathrm{PEOT}}$—average molecular weight of PEOT segment; *M*_n_, *M*_w_—number and weight average molecular weight determined by SEC according to ref. [30].

**Table 2:** Thermal properties of PTT-*block*-PEOT copolymers.

Sample	PEOT Segment						PTT Segment					
	*T*_g1_	*T*_m1_	∆*H*_m1_	*T*_c1_	∆*T*_PEO_	$x_{c}^{\mathrm{PEO}}$	*T*_m2_	∆*H*_m2_	*T*_c2_	∆*H*_c2_	∆*T*_PTT_	$x_{c}^{\mathrm{PTT}}$
	°C	°C	J/g	°C	°C	%	°C	J/g	°C	J/g	°C	%
50/50P1	−45	-	-	-	-	-	195	27.4	125	27.5	70	18.8
40/60P1	−47	-	-	-	-	-	177	20.6	93	20.6	84	14.1
30/70P1	−48	7	6.3	7	0	3.2	149	13.5	58	13.7	91	9.2
50/50P2	−49	15	22.9	-27	42	11.6	212	34.0	167	34.4	45	23.3
40/60P2	−48	20	30.1	-10	30	15.3	196	21.5	140	23.1	56	14.7
30/70P2	−49	28	50.9	4	24	25.8	178	14.6	126	14.5	52	10.0
50/50P3	−48	26	32.5	17	9	16.5	218	33.8	143	33.6	75	23.1
40/60P3	−48	30	45.3	4	26	23.0	211	22.8	133	22.6	78	15.6
30/70P3	−48	37	68.1	-10	47	34.6	195	19.5	139	19.9	56	13.6

*T*_g1_—glass transition temperature of soft phase; *T*_m1_ and, *T*_c1_—melting and crystallization temperature of PEO segments respectively; Δ*H*_m1_—enthalpy of melting of PEO crystals; $x_{c}^{\mathrm{PEO}}$—content of PEO crystals in the sample; *T*_m2_ and, *T*_c2_ melting and crystallization temperature of PTT segment; Δ*H*_m2_ and ΔH_c2_—enthalpy of melting and crystallization of PTT crystals; $x_{c}^{\mathrm{PTT}}$—content of PTT crystals in the sample; **∆***T*_PEO_ = *T*_m1_–*T*_c1_—supercooling for PEO crystallization; **∆***T*_PTT_ = *T*_m2_–*T*_c2_—supercooling for PTT crystallization.

**Table 3:** Tensile properties of PTT-*b*-PEOT copolymers.

Sample	*E*	σ_b_	ε_b_
	MPa	MPa	%
50/50P1	134.2 ± 2.3	21.70 ± 0.54	533 ± 14
40/60P1	87.1 ± 0.9	17. 56 ± 0.81	582 ± 16
30/70P1	42.5 ± 1.1	12.78 ± 1.07	685 ± 24
50/50P2	99.2 ± 0.9	25.43 ± 0.21	841 ± 22
40/60P2	77.4 ± 2.2	20.65 ± 0.91	940 ± 16
30/70P2	44.5 ± 0.4	19.33 ± 0.47	948 ± 36
50/50P3	86.5 ± 0.8	29.11 ± 1.17	877 ± 5
40/60P3	128.3 ± 2.1	26.41 ± 0.41	938 ± 25
30/70P3	164.2 ± 2.1	23.52 ± 0.32	921 ± 11

*E*—tensile modulus; σ_b_—stress at break; ε_b_—strain at break.
